# The History of Tuberculosis

**Published:** 1885-04

**Authors:** Albert Johne


### Art. XV.—THE HISTORY OF TUBERCULOSIS.

BY DR. ALBERT JOHNE.

[Continued from page 350, Vol. 5.]

### The Present Views as to Tuberculosis in Man and Animals as an Infectious Disease.

Anatomically speaking, tuberculosis may be looked upon as a destructive inflammatory process which leads to the development of small cellular, non-vascular, granular nodes, that sooner or later undergo cheesy degeneration. This inflammatory process is clinically characterized by its strongly progressive nature. It occurs not only in the organ primarily complicated, but disseminates over the entire organism, and is also transmissible from man to animals as well as from one animal to another, or in other words, it has been proven beyond all question that tuberculosis is an infectious disease, that is completely identical in its characteristics both in man and animals.

No one has better described tuberculosis, from this point of view, than Cohnheim, who does not look upon the purely anatomical nature of the tubercle as an absolutely necessary diagnostic point, but rather that these neoplasms have a common origin wherever found. Neither the cellular character of the nodes, nor the caseation once considered so essential an attribute, can be looked upon as decisive, neither being found *only* in such connection. They can only belong to tuberculosis when by inoculation they produce it. In most cases of natural infections the inficiens finds access to the organism by means of the respiratory track, the lungs being the first to become diseased, and from thence the processes extend over the body. By means of expectoration infectious materials become transported to the trachea and larynx, and by swallowing the same to the intestinal track. In other cases the latter can become infected through food, when the intestinal organs are the primary seat of disease. In such cases the lymphatic organs of the mouth and pharynx frequently become diseased very early in its course with extension to the superior lymphatics of the neck. The œsophagus and stomach often escape attack on account of the rapid passage of the infectious material through these organs, while extensive disease may occur in the deeper seated portions of the canal which extend to the glandular organs of the abdominal cavity as well as the fallopian tubes and uterus. As the inficiens of tuberculosis is excreted by means of the kidneys, these organs frequently become the seat of its peculiar processes, which extend to the ureters, bladder, prostate and testicles. In consequence of infection by means of coitus, the above organs may also be complicated, the inficiens passing inward along the urinary canal.

The condition of infection seems to exactly correspond in man and animals. It matters not whether the infectious material be derived from either the one or the other species, or whether it takes place accidentally or *per intentionem.* This complete correspondence in the manner in which infection occurs in both man and animals adds a strong link to the chain in the identity of the five varieties of the disease.

The dispersion of the inficiens over the organism results in a variety of ways. It has been demonstrated, for a long time, that the disease extends by continuity along the lymph tracks in the interstitial tissue and serous membranes. The manner in which generalization takes place has been described in detail by Ponfick and Weigert. According to these authors, it occurs when the inficiens gains access to the circulation, and is thus carried to all parts of the body.

Ponfick has shown, in these cases, that the *atrium* was to be seen in ulcerations and infiltrations in the thoracic duct; Weigert has demonstrated similar conditions in the pulmonary vein. Lately, Weigert has still more sharply defined the generalization of miliary tuberculosis, and understands thereby the eruption of tubercles in such places that are very accessible to the action of the inficiens by means of the circulation, and excludes the extension of the disease by continuity as belonging to the processes of generalization.

Orth does not admit of any distinction between chronic tuberculosis or phthisis due to caseous pneumonia and acute miliary tuberculosis from the genetic point of view, and explains the microscopic difference in these conditions by assuming that when the infectious elements gain access to the circulation in large quantities, or gradually and in small quantities at a time, or when its action is more of a local character, or whether the individual is more or less resistant to infections, so do the anatomical results have an acute miliary character, or caseous with or without nodular developments.

The peculiar nature of the inficiens in tuberculosis has, until lately, been a matter of discussion and doubt.

The views of Buhl, Rindfleisch, and other clinicians, that the organism itself produced the inficiens, have been satisfactorily contradicted. The clinical fact that a quantity of caseous-necorbic material can be absorbed without tuberculosis following, clearly shows that all such elements do not have specific action, and that, in addition, it is necessary that a specific infectious element be introduced to such material.

Cohnheim says that "only such hyperplastic or inflammatory products which undergo specific, *i. e.*, infectious, tuberculous caseation can be a product of the tuberculous inficiens." A pleuritis which is not followed by absorption, but continues, or relapses, and finally leads to pulmonary tuberculosis, is from the beginning of a specific nature. The same is true of a bronchitis, pneumonia, or hypertrophied lymph gland, which undergo caseation, as they are caused, at first, by a tuberculous, scrofulous virus. Or, as Zeigler expresses himself with reference to the relations of tuberculosis to scrofulosis, "the caseated inflammatory product of scrofulosis is not, therefore, infectious because scrofulous, but because it is already tuberculous, or the inflammatory product has become secondarily infected."

All the clinical facts which have been thus far collected speak most strongly for an organized virus, *i. e.*, one capable of auto-multiplication in a diseased organism as the cause of tuberculosis.

Zuru, 1871, had reported the presence of micrococci in the blood of a cow diseased with tuberculosis; when placed in water they demonstrated an individual movement; the addition of carbolic acid to the preparation caused this movement to cease. Zuru found the same micrococci to be present in the tissue at a point of inoculation as well as in miliary tubercles in the lungs. Chauveau, 1872, discovered corpuscular parasites in tuberculous material; Klebs, 1873, defended a similar opinion. Continued experimentation has given a stronger foundation to these observations. Koch has the credit of removing them from a hypothetical to an exact scientific foundation. In the early part of 1881, Koch, and in 1882, Ehrlich and Baumgarten demonstrated, beyond all doubt, the presence of peculiar bacilli in tuberculous material. Koch and others were soon enabled to cultivate these germs in a pure state, and to successfully produce tuberculosis in animals devoted to inoculation experiments. The bacilli of tuberculosis are described by Koch as nearly filliform, their length being from a half to the whole diameter of a red blood cell. They are frequently found in large masses with the so-called giant cells. These bacilli of Koch distinguish themselves from other fission fungi that have heretofore been connected with tuberculosis by the specific manner with which they become stained, and the fact that they are also immovable and require a long time to multiply.

Koch has been enabled to carry them through numerous pure cultivations, and then to produce general miliary tuberculosis in Guinea-pigs, rabbits, rats, mice, dogs and cats by inoculation. It mattered not whether the original material was derived from man or animals, the results were always the same, and corresponded exactly with those obtained by other investigators.

Koch considered the bacilli to be such a characteristic element in tuberculosis that he declared that, with the demonstration of the presence, it was possible to draw the limits as to what was or was not tuberculosis in a more certain manner than we had ever before been able to do. In the future this difficulty will no longer trouble either pathologists or clinicians. Cohnheim and Koch both agree that it is not the anatomical construction, but the etiological proof of the presence of these specific bacilli that will be the criterion in the future as to what is tuberculosis.

Assuming this opinion to be correct, we must consider Miliary Tuberculosis, Caseous Pneumonia and Bronchitis, Intestinal and Glandular Tuberculosis, and Perlzucht of Cattle, as well as Spontaneous and Inoculable Tuberculosis in animals as completely identical processes. At least, it is a fact, that the greater part, *if not all*, of scrofulous and joint diseases should be looked upon as true tubercular.

As to the *when and how* these parasites gain access to the organism, Koch has shown that the bacilli of tuberculosis require to their development, a temperature of from 30 to 41° C, and that their development is of a very protracted nature in comparison. As in our climate opportunities for a continued temperature of 30° C for a period of two weeks, the time these fungi require to develop, are not given outside of the animal organism, it is evident that they can only come to development within an animal body.

As by far the greater number of cases of tuberculosis have their origin in the respiratory organs, it is probable that the bacilli gain access with the inspired air. Their immediate origin is easy of discovery. In consequence of the innumerable masses of tubercle bacilli which collect in the caverns in the lungs of phthisical patients, there can be no doubt that they are expectorated with the sputum and subjected to desiccation. The length of time that such material can retain its infectious qualities is shown by inoculations where sputum that has been carefully desiccated for eight weeks caused tuberculosis in Guinea-pigs with the same certainty as fresh material. We are accordingly justified in assuming that earth, floors, clothing, bedding, etc., thus infected can retain its infectious characteristics for a long time, and when it becomes converted to dust and taken up by the air, give rise to tuberculosis in susceptible organisms for a long time.

The infection of the organism by tubercle bacilli is not possible from all kinds of small wounds of the surface, as is the case with the rapidly multiplying bacilli of anthrax. On account of their protracted development it is necessary that they remain fixed upon a nutrient material, and in a protected position, for a long time, otherwise they are easily eliminated. The infectious elements must be deeply introduced to insure inoculation on this account. In the lungs it is the same, stagnating secretions, the loss of epithelium, and deep-seated cavities forming the best seat for their development.

Tuberculosis can only be successfully combatted by putting an end to the means of infection. The diseased organism is to be looked upon as the centre for infection. Two things must be looked upon as the seat of infection from this stand-point: 1st. The sputum from phthisical persons, and substances polluted with the same; 2d. Tuberculosis of animals.

The Etiological Connection between Human and Animal Tuberculosis.—With the proof which Koch has given of the identity and virulence of both human and animal tuberculosis, this question has assumed a prominent place in the researches of experimental pathologists, viz: Is tuberculosis transmissible from animals to animals, and from them to man, by means of the consumption of flesh and milk from animals having this disease? Gerlach was the first to answer this question in the affirmative. In the year 1875 he considered that he had established the identity of the two diseases, and said: "We have, therefore, not only the right, but the duty, to adapt the result of our investigations to questions of public health. They point to one cause of tuberculosis in man as being in the food we eat."

Zuru, Semmer, Toussaint also held the flesh of tuberculous animals as unconditionally infectious (although to a less degree than genuinely infected material). As tubercles are, however, developed in the interstitial tissue of flesh, as has been demonstrated by Schutz and Leisering, the infections of such meat will naturally depend upon the degree of development of tubercles in the same. Cooking destroys this quality in small pieces, but not in large ones. All other investigators in this important question have adopted similar views.

Klebs takes a more positive position with reference to the flesh of cattle thus diseased, and especially emphasizes the dangerous character of milk from tuberculous cows, its infectiousness depending upon the degree of extension which the disease has acquired in such animal. The inficiens is to be sought in the milk-source in a suspended condition, and is not destroyed by the general manner of cooking. Flemming, Rivolta, Perroncito and Bollinger have all adopted similar views, and endorsed the position assumed by Gerlach. Bollinger considers the danger of transmission to human beings as being greater from the consumption of the milk from such diseased animals than from that of the flesh, as the former is more frequently taken in an uncooked condition, especially by children. The receptivity of the latter for the injurious influences of food far exceeds that of matured individuals. Bollinger even goes so far as to assume that the influence which such food has exerted upon babes may have too often been attributed to hereditary conditions. He also places emphasis upon the general disposition which seems to exist in man to tuberculosis. Bollinger seeks further to support his position by quoting the frequency with which tubercular phthisis is quoted as *causa lethalis* in human statistics: 30.25 per cent. of all deaths in Munich, and according to Koch, one-seventh of all human deaths, are due to phthisis.

As, according to Adam's and Goring's statistics, tuberculosis in cattle seems to increase as age advances, it seems advisable to subject all cows, especially those over six years of age, to a most critical examination. Clinical cases in human beings of tuberculosis traceable to infection from animal products, have, as yet, not been conclusively proven. Kleuk and Zippelus report such a case, while Dr. Stang in Amerbach reports a case of tuberculosis in a five-year-old child, where no trace of an hereditary influence could be discovered, which had been for a long time fed upon warm milk taken directly from a cow in which tuberculosis was unquestionably proven to exist.

Bollinger, however, has failed to notice quite a number of cases of a similar nature: for instance, Demone, Uffelmann, Ebstein, Hergard, Felizst, have reported such. Zippelus has endeavored to gather statistics with reference to the occurrence of tuberculosis in cows and babes. This important subject needs much more detailed consideration than it has thus far received from Boards of Health. According to Z.'s table, it appears that the death-rate among children under two years old, from tuberculosis, is the greatest in those regions where meat inspection has shown the greatest prevalence of tuberculosis in slaughtered cattle.

Orth also considers the transmission of bovine tuberculosis to human beings as possible, both through consumption of flesh and milk. Cohnheim and Aufrecht both consider the milk of tuberculous cows to be a cause of phthisis mesenterica, the primary intestinal and acute miliary tuberculosis of children. Semmer adopts Bollinger's views. The transmission of tuberculosis to dogs by such food, speaks strongly for these views, for dogs have as little susceptibility to this disease as any animal. Virchow is inclined to the same opinion, though he does not consider that the results of the experiments that have thus far been made fully warrant condemning the meat of such cattle from consumption, as tubercles have not yet been proven to exist in the flesh itself, though the parts infected should not be permitted to be sold.

Toussaint believes that infection results easier when tuberculous material is taken into the intestines than when inoculated subcutaneously. Basing his ideas upon exact experiment, he considers it dangerous to feed meat or its juices, either raw or half-cooked, to invalids or children.

When we carefully consider all the evidence, *pro* or *con*, in this question, as well as the results of Koch's investigations, which absolutely prove the identity and virulence of the disease in both man and animals, we are forced to the conviction that we can no longer doubt the possibility of the transmission of tuberculosis from animals to man through the consumption of the flesh and milk of animals thus diseased. Further investigations must be made in the most exact manner by unquestionably competent persons. Until it has been positively demonstrated that such a possibility does not exist, we must accept the evidence of Gerlach, Chauveau, Klebs and Toussaint with reference to the transmissibility of bovine tuberculosis, and see that the products from animals thus diseased are not sold for human food.

The exact amount of danger, and under what circumstances it is present in such food, is still an open question, as well as the best means to destroy the inficiens so as to make it inactive. We at present know that boiling heat can destroy its virulency, but even cooking for a half an hour is not always sufficient for the heat to penetrate through large pieces of meat. Milk is naturally more securely and easily treated.

(*To be continued.*)

